# Prevalence of Metabolic Syndrome in Patients With Psoriasis Vulgaris: A Hospital-Based Cross-Sectional Study

**DOI:** 10.7759/cureus.68037

**Published:** 2024-08-28

**Authors:** Fatma Etgü, Emine Dervis

**Affiliations:** 1 Dermatology, Ordu University, Ordu, TUR; 2 Dermatology, Emine Dervis Klinik, Istanbul, TUR

**Keywords:** cardio vascular disease, dyslipidemia, obesity, diabete mellitus, abdominal circumference, hypertension, prevalence‎, metabolic syndrome (mets), psoriasis comorbidities, psoriasis vulgaris

## Abstract

Introduction

Psoriasis is a chronic, recurrent inflammatory skin condition that affects 1-3% of the global population. Increasing evidence suggests a higher prevalence of metabolic syndrome (MetS) among individuals with psoriasis. This study aims to investigate the prevalence of MetS in patients with psoriasis and compare the findings with existing literature.

Methods

This cross-sectional, hospital-based study included 311 patients with psoriasis. Data were retrospectively collected from hospital records.

Results

The study included 311 patients with psoriasis (144 females and 167 males), with a mean age of 41.6 years (range 18-87). The mean BMI was 27.13 ± 5.29 kg/m², and the average waist circumference was 93 cm. Mean fasting blood sugar levels were 100 mg/dL, mean high-density lipoprotein (HDL) cholesterol was 44 mg/dL, and mean triglycerides were 132 mg/dL. MetS was diagnosed in 60 patients (19.3%). Patients with MetS had significantly higher mean waist circumference, higher rates of hypertriglyceridemia, hyperglycemia, and hypertension, and lower mean HDL levels (p < 0.05). There was no significant association between MetS and psoriasis severity, disease duration, family history, smoking, or alcohol consumption habits.

Conclusions

In this study, the prevalence of MetS among patients with psoriasis was 19.3%. MetS prevalence was not linked to smoking status, alcohol consumption, family history of psoriasis, disease duration, or severity. It is crucial for dermatologists treating psoriasis patients to be aware of MetS, its components, and associated cardiovascular risks.

## Introduction

Psoriasis is a prevalent chronic, recurrent inflammatory skin disease characterized by sharply demarcated, scaly erythematous plaques, affecting 1-3% of the global population [1,2]. While traditionally viewed as a purely cutaneous condition, psoriasis is now recognized as a systemic disease involving increased inflammatory activity that impacts multiple organs, especially the skin and joints [1,3]. This systemic involvement is linked to numerous comorbidities, categorized into classic, emerging, lifestyle-related, and treatment-related types [4,5]. Among these, metabolic syndrome (MetS), its components, and cardiovascular diseases are most commonly encountered [4,6]. MetS comprises several metabolic disorders, including abdominal obesity, insulin resistance, hyperglycemia, atherogenic dyslipidemia (characterized by hypertriglyceridemia and low high-density lipoprotein (HDL) levels), and hypertension (HT). Each component is associated with cardiovascular diseases and diabetes mellitus [7]. Increasing evidence suggests a higher prevalence of MetS among psoriasis patients, often in a dose-dependent manner [8,9]. This study aimed to investigate the prevalence of MetS in patients with psoriasis and compare the findings with existing literature.

## Materials and methods

This hospital-based cross-sectional study was conducted at the Dermatology Outpatient Clinic of Haseki Training and Research Hospital, Istanbul, Turkey, between 2012 and 2013. Data for patients with psoriasis were retrospectively gathered from hospital records. The inclusion criteria were being over 18 years of age, having a clinical diagnosis of psoriasis for at least six months, and not receiving any psoriasis treatment for at least one month before enrollment.

The collected data included age, gender, height, weight, BMI, waist circumference, blood pressure, psoriasis severity, duration of psoriasis, smoking habits, alcohol consumption habits, and any drug treatments for HT, diabetes, or hyperlipidemia. Psoriasis severity was assessed using the Psoriasis Area and Severity Index (PASI), with scores greater than 10 indicating severe disease. BMI was calculated as weight divided by height squared (kg/m²). Waist circumference was measured at the midpoint between the lower border of the rib cage and the top of the iliac crest using a non-stretchable tape. Blood pressure was measured twice at 30-minute intervals with the patient seated for five minutes; the mean of these two measurements was recorded.

Venous blood samples, collected after at least eight hours of overnight fasting, were analyzed for fasting blood sugar (FBS) (normal range: 75-105 mg/dL), HDL cholesterol (normal range: 40-80 mg/dL), low-density lipoprotein cholesterol (normal range: 85-125 mg/dL), total cholesterol (normal range: 110-200 mg/dL), and triglyceride (TG) levels (normal range: 50-200 mg/dL). MetS was diagnosed based on NCEP ATP III criteria, requiring at least three of the following five conditions: abdominal obesity (waist circumference ≥102 cm in men and ≥88 cm in women), serum TG ≥150 mg/dL or medication for elevated TG, HDL cholesterol <40 mg/dL in men and <50 mg/dL in women or medication for low HDL cholesterol, blood pressure ≥130/85 mmHg or medication for elevated blood pressure, and FBS ≥100 mg/dL or medication for elevated blood glucose.

Smoking status was classified as never smokers, ex-smokers, or current smokers. Alcohol consumption was categorized as none or regular drinkers.

Statistical analysis was performed using IBM SPSS Statistics for Windows, Version 21.0 (Released 2012; IBM Corp., Armonk, NY, USA). Categorical parameters were expressed as counts and percentages, while continuous parameters were reported as mean and standard deviation values. Data distribution was assessed using the Kolmogorov-Smirnov test. The chi-square test was used for categorical variable analysis, and the independent-sample t-test was used for quantitative variable analysis. A p-value of <0.05 was considered statistically significant, with a 95% CI.

## Results

The study included 311 patients with psoriasis (144 females and 167 males), with a mean age of 41.6 ± 14.78 years (range 18-87). The mean BMI was 27.13 ± 5.29 (Table 1). The disease duration was less than 10 years for 47% of the patients, 11-20 years for 29%, and more than 21 years for 24%. Smoking habits were reported as follows: 30.9% of patients had never smoked, 49.5% were active smokers, and 19.6% were ex-smokers. PASI scores were below 10 in 63.3% of patients, indicating non-severe disease, while 36.7% had severe psoriasis. A positive family history of psoriasis was noted in 30% of patients.

**Table 1:** Demographic and anthropometric characteristics of the study population BMI (kg/m²) (mean ± SD): 27.13 ± 5.29

Variable	n	%
Gender		
Male	167	53.70%
Female	144	46.30%
BMI (kg/m²)		
<18.5 (underweight)	3	1.20%
18.5-24.9 (normal)	82	32.70%
25-29.9 (overweight)	90	35.90%
30-39.9 (obesity)	67	26.70%
≥40 (massive obesity)	9	3.60%

The average waist circumference was 93 cm, with 38.6% of patients exceeding normal values. The mean FBS level was 100 mg/dL, with 17.7% of patients having elevated levels. The mean HDL cholesterol was 44 mg/dL, and 54% of patients had low HDL levels. The mean TG level was 132 mg/dL, with 28.9% of patients having elevated levels. HT was present in 13.5% of patients. MetS was diagnosed in 60 patients (19%) with psoriasis. Table 2 provides data on the prevalence of MetS and its components.

**Table 2:** Prevalence of MetS and its components FBS: ≥100 mg/dL; HDL cholesterol: <40 mg/dL in men and <50 mg/dL in women; HT: ≥130/85 mmHg; TG: ≥150 mg/dL; waist circumference: ≥102 cm in men and ≥88 cm in women FBS: fasting blood sugar; HDL: high-density lipoprotein; HT: hypertension; MS: metabolic syndrome; TG: triglyceride

Component	Number	%
Waist circumference		
High	97	38.60%
Normal	154	61.40%
HT		
Present	42	13.50%
Absent	269	86.50%
FBS		
High	53	17.70%
Normal	246	82.30%
HDL		
Low	168	54%
Normal	143	46%
TG		
High	90	28.90%
Normal	221	71.10%
MetS		
Present	60	19.30%
Absent	251	80.70%

A comparison of patients with and without MetS revealed that those with MetS had a higher mean waist circumference, greater rates of hypertriglyceridemia, hyperglycemia, and HT, and lower mean HDL levels (p < 0.05). There were no significant differences in smoking or alcohol consumption between the two groups. Additionally, the presence of MetS was not associated with psoriasis severity, disease duration, mean PASI score, or positive family history.

## Discussion

Epidemiological studies have shown that the prevalence of MetS and associated conditions - such as HT, hyperlipidemia, obesity, diabetes, and cardiovascular disease - are more common in patients with psoriasis compared to controls [10]. Physical inactivity, smoking, alcohol consumption, obesity, HT, and hyperlipidemia are modifiable factors that contribute to MetS and cardiovascular mortality. Psoriasis patients are particularly prone to these risk factors due to social isolation, psoriatic arthritis-related pain, increased predisposition to depression, physical inactivity, and unhealthy eating habits [4,11]. Early detection of MetS and its components in patients with psoriasis, along with appropriate and continuous lifestyle changes, is crucial for preventing cardiovascular diseases, which are a leading cause of mortality in these patients [12].

In this study, the prevalence of MetS among psoriasis patients was 19.3%. Previous studies have reported varying prevalence rates of MetS in psoriasis patients: 30.1% [7], 4.2% [8], 32.8% [11], 10.3% [13], 35.5% [14], 14.1% [15], 24.6% [16], 53.0% [17], 14.3% [18], and 49.4% [19]. The discrepancy in findings may be attributed to differences in sample populations, racial variations, and diagnostic criteria for MetS. For example, a study by Kim et al. in Korea found no significant correlation between psoriasis and MetS [13]. Similarly, Mebazaa et al. reported that while MetS was more common in the psoriasis group compared to controls, the difference was not statistically significant [14]. Chen et al. and Damevska et al. also found no significant difference in MetS prevalence between psoriasis and control groups [15,16]. Conversely, other studies have documented a significantly higher prevalence of MetS in psoriasis patients [11,17-19]. These differences may arise from genetic factors, racial and ethnic variations, lifestyle habits, and varying diagnostic criteria for MetS.

While Gisondi et al. and Meziane et al. identified age as a risk factor for MetS [20,21], our study did not find an association between age and MetS. Additionally, we observed no association between the severity of psoriasis and MetS, which is consistent with several other studies [13,22]. In contrast, Sommer et al. found a correlation between MetS prevalence and psoriasis severity, and Langan et al. reported a dose-dependent relationship between MetS and psoriasis [8,23]. Zindanci et al. also found that MetS prevalence was not linked to the duration or severity of psoriasis or to smoking status [17].

Our study revealed that more than one-third of psoriasis patients had a higher waist circumference, consistent with previous studies that have highlighted a higher prevalence of abdominal obesity among psoriasis patients compared to healthy controls [8,20,22]. Factors such as social isolation, physical inactivity, depression, and unhealthy eating habits may contribute to obesity in psoriasis patients, negatively impacting daily life and quality of life. Central obesity is a significant factor in MetS development, as adipocytes produce various proinflammatory cytokines [15,23].

In the current study, a significant difference was observed in mean BMI between participants with and without MetS, highlighting the critical role of obesity in the development of MetS. This finding contrasts with some previous studies [24], as many authors have reported that any increase in BMI correlates with a higher prevalence of psoriasis [14,25,26]. Specifically, Zindanci et al. found that BMI significantly increased only among female psoriasis patients [17].

Many studies have reported a higher prevalence of HT among psoriasis patients compared to controls [8,11,17,22,23], independent of shared risk factors such as obesity, smoking, and a sedentary lifestyle. However, the underlying mechanism linking HT and psoriasis requires further investigation. In our study, only 13.5% of psoriasis patients had HT. Gisondi et al. and Ferdinando et al. did not find a significant difference in HT prevalence between psoriasis patients and healthy controls [19,20].

Our study supports previous findings linking psoriasis with hyperglycemia, as 17.7% of patients had elevated FBS levels [11,18,19]. Many studies have found no significant difference in hypertriglyceridemia between psoriasis and control groups [20,27,28], while others have shown a higher prevalence of hypertriglyceridemia in psoriasis patients [11,18,21,22]. Langan et al. also reported a dose-dependent relationship between hypertriglyceridemia and psoriasis [8].

We observed low HDL levels in 54% of psoriatic patients, which aligns with previous studies [22]. Chen et al. found lower HDL levels in psoriasis patients compared to controls, although the difference was not statistically significant [15]. Given HDL’s role in anti-atherogenic activities and endothelial maintenance, low HDL levels are associated with cardiovascular events in psoriasis patients with MetS [20,29].

Smoking is a known risk factor for MetS and has been linked to psoriasis severity [20,23,27]. Despite nearly half of the patients being active smokers, there were no significant differences in smoking or alcohol consumption habits between psoriasis patients with and without MetS. Our findings align with previous studies that found no association between gender and MetS [14,17], although Adışen et al. reported higher MetS prevalence among female psoriasis patients [26].

This study has several limitations. Firstly, it was conducted in a tertiary hospital setting, so the results may not be generalizable to all psoriasis patients. Secondly, due to the cross-sectional design, causality cannot be established. Lastly, the sample size was relatively small.

## Conclusions

In this hospital-based cross-sectional study, we observed that 19.3% of psoriasis patients had MetS. The prevalence of MetS did not correlate with smoking status, alcohol consumption, family history of psoriasis, disease duration, or disease severity. Our findings confirm that patients with psoriasis have an elevated risk of MetS and associated cardiovascular complications, with risk levels potentially increasing with disease severity. Consequently, it is crucial to diagnose MetS and its components promptly and to promote lifestyle changes such as weight reduction, increased physical activity, healthy eating habits, smoking cessation, and alcohol reduction. Dermatologists should be vigilant about the risks associated with MetS, prioritize early detection, offer comprehensive patient education, and facilitate timely referrals when needed.
